# CXCR4 and CXCR7 transduce through mTOR in human renal cancer cells

**DOI:** 10.1038/cddis.2014.269

**Published:** 2014-07-03

**Authors:** C Ieranò, S Santagata, M Napolitano, F Guardia, A Grimaldi, E Antignani, G Botti, C Consales, A Riccio, M Nanayakkara, M V Barone, M Caraglia, S Scala

**Affiliations:** 1 Istituto Nazionale per lo Studio e la Cura dei Tumori, Fondazione “Giovanni Pascale”—IRCCS—ITALY, Naples, Italy; 2 Department of Biochemistry, Biophysics and General Pathology, Second University of Naples, Naples, Italy; 3 Department of Translational Medical Science and European Laboratory for the Investigation of Food Induced Disease (ELFID), University of Naples, Federico II, Italy

## Abstract

Treatment of metastatic renal cell carcinoma (mRCC) has improved significantly with the advent of agents targeting the mTOR pathway, such as temsirolimus and everolimus. However, their efficacy is thought to be limited by feedback loops and crosstalk with other pathways leading to the development of drug resistance. As CXCR4–CXCL12–CXCR7 axis has been described to have a crucial role in renal cancer; the crosstalk between the mTOR pathway and the CXCR4–CXCL12–CXCR7 chemokine receptor axis has been investigated in human renal cancer cells. In SN12C and A498, the common CXCR4–CXCR7 ligand, CXCL12, and the exclusive CXCR7 ligand, CXCL11, activated mTOR through P70S6K and 4EBP1 targets. The mTOR activation was specifically inhibited by CXCR4 antagonists (AMD3100, anti-CXCR4-12G5 and Peptide R, a newly developed CXCR4 antagonist) and CXCR7 antagonists (anti-CXCR7-12G8 and CCX771, CXCR7 inhibitor). To investigate the functional role of CXCR4, CXCR7 and mTOR in human renal cancer cells, both migration and wound healing were evaluated. SN12C and A498 cells migrated toward CXCL12 and CXCL11; CXCR4 and CXCR7 inhibitors impaired migration and treatment with mTOR inhibitor, RAD001, further inhibited it. Moreover, CXCL12 and CXCL11 induced wound healing while was impaired by AMD3100, the anti CXCR7 and RAD001. In SN12C and A498 cells, CXCL12 and CXCL11 promoted actin reorganization characterized by thin spikes at the cell periphery, whereas AMD3100 and anti-CXCR7 impaired CXCL12/CXCL11-induced actin polymerization, and RAD001 treatment further reduced it. In addition, when cell growth was evaluated in the presence of CXCL12, CXCL11 and mTOR inhibitors, an additive effect was demonstrated with the CXCR4, CXCR7 antagonists and RAD001. RAD001-resistant SN12C and A498 cells recovered RAD001 sensitivity in the presence of CXCR4 and CXCR7 antagonists. In conclusion, the entire axis CXCR4–CXCL12–CXCR7 regulates mTOR signaling in renal cancer cells offering new therapeutic opportunities and targets to overcome resistance to mTOR inhibitors.

Renal cell carcinoma (RCC) is the most lethal malignancy among urological cancers with a total of 64 770 new cases and 13 570 deaths estimated in the United States in 2012.^1^ A growing understanding of the molecular biology of RCC changed the therapeutic approach toward target-based agents. Since 2005, the US Food and Drug Administration (FDA) has approved six new target agents for metastatic RCC that antagonize two principal signaling pathways: the vascular endothelial growth factor receptor (VEGF) and the mammalian target of rapamycin (mTOR).^2^ The mTOR is an atypical intracellular serine/threonine protein kinase regulated by phosphatidylinositol 3-kinase (PI3K).^3^ mTOR exists in two distinct complexes termed mTOR complex 1 (mTORC1) comprising mTOR, mLST8 (also termed G-protein *β*-subunit-like protein, G*β*L, a yeast homolog of LST8), raptor (regulatory associated protein of mTOR) and PRAS40 (proline-rich Akt substrate, 40 kDa), and mTOR complex 2 (mTORC2) comprising mTOR, mLST8, rictor (rapamycin-insensitive companion of mTOR), mSin1 (mammalian stress-activated protein kinase (SAPK)-interacting protein 1), protor (protein observed with rictor) and PRR5 (proline-rich protein 5).^4^ mTORC1 responds to amino acids, stress, oxygen, energy and growth factors and is sensitive to rapamycin; when active, mTORC1 promotes cell growth and also drives cell-cycle progression. Alternatively, mTORC2 regulates cytoskeletal organization and cell survival/metabolism and is sensitive to rapamycin over longer incubation times or at higher doses.^3^ mTORC1 controls cell growth and translation through the phosphorylation of ribosomal protein S6 kinase (S6K) and of eukaryotic translation initiation factor 4EBP1, which regulate either the translation of ribosomal proteins or the cap-dependent translation by inhibition of eukaryotic translation initiation factor 4E, respectively.^3, 4^ The activated mTOR pathway has been identified in several human malignancies, thus being an attractive target for anticancer therapy. mTORC1 activity is inhibited by rapalogs such as rapamycin (sirolimus) and associated analogs (temsirolimus/CCI-779, RAD001, ridaforolimus/AP23573).^5^ These drugs suppress mTORC1 activity forming a complex with FK506-binding protein 12. Temsirolimus (rapamycin analog) was the first mTOR inhibitor approved as first-line treatment in patients with poor-prognosis metastatic RCC (mRCC) patients,^3^ ridaforolimus is currently tested in phase III clinical trials^5^ and RAD001 is indicated as second-line treatment in patients with RCC at failure of first-line treatment with sunitinib or sorafenib. Other indications are subependymal giant cell astrocytoma associated with tuberous sclerosis and progressive neuroendocrine tumors of pancreatic origin.^5^ Although mTOR inhibitors prolong progression-free survival in patients with advanced RCC, most patients develop resistance to mTOR-inhibiting agents, limiting their efficacy; the new frontier of inhibiting the mTOR pathway is to identify agents targeting the feedback loops and crosstalks with other pathways involved in the acquired resistance to mTOR inhibitors.^6^

Chemokines and their receptors have been implicated in regulating RCC growth, angiogenesis and metastases.^7^ In RCC, VHL mutation resulted in HIF-dependent CXCR4 activation^8^ and CXCR4 expression predicted poor tumor-specific survival.^8, 9, 10^ Recently, CXCL12 was shown to bind with high affinity the orphan receptor CXCR7/RDC1, which also binds a second ligand in the form of interferon-inducible T-cell *α* chemoattractant (I-TAC/CXCL11).^11^ Whereas the CXCR4 activity is primarily G-protein-mediated, CXCR7 is considered an atypical GPCR because ligand binding does not result in intracellular Ca2+ release.^11^ Some studies provided evidence that CXCR7 represents a ‘decoy' receptor, which is responsible for either sequestering extracellular CXCL12^12^ or modulating CXCR4 signaling by forming CXCR7–CXCR4 heterodimers.^13^ In contrast, others demonstrated that CXCR7 relays intracellular signals^14, 15, 16, 17^ and promotes cell motility^18, 13, 19^ acting through *β*-arrestin.^20, 21^ CXCR7 is highly expressed in human cancers such as prostate, lung, glioma, ovarian, breast cancer cells and in tumor-associated blood vessels and seems to be essential for survival, adhesion and growth of tumor cells.^11, 14, 15, 22, 23, 24^ It was recently demonstrated that CXCR4 and CXCR7 predict prognosis in RCC.^10, 25^ CXCL12 activates CXCR4 and the derived signaling can transduce on the mTOR pathway in pancreatic cancer, gastric cancer and T-cell leukemia cells;^26, 27, 28, 29^ antagonists targeting PI3K and/or mTOR inhibited CXCL12-mediated cell migration and this effect was primarily attributed to the inhibition of mTORC1 and consequent decrease in RhoA, Cdc42 and Rac1 in human gastric carcinoma cells.^28^

Aim of the study was to evaluate interactions between the CXCL12–CXCR4–CXCR7 axis and the mTOR pathway in human renal cancer cells to identify new therapeutic opportunities and overcome resistance mechanisms.

## Results

### CXCL12–CXCR4 activates mTOR signaling in human renal cancer cells

**Figure 1:**
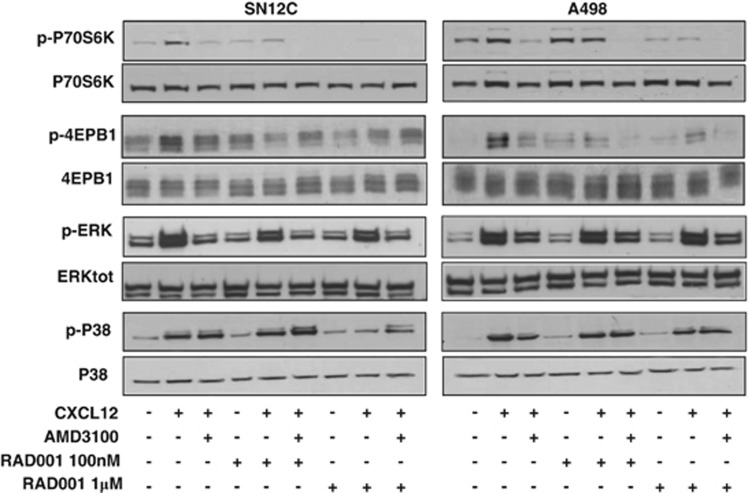
CXCL12 activates mTOR signaling through CXCR4 in RCC. The SN12C and A498 human renal cancer cell lines were stimulated with CXCL12 (100 ng/ml) and p-P70S6 kinase, p-4EBP1, p-ERK1/2 and p-P38 proteins were measured in absence or presence RAD001(100 nM and 1 *μ*M) using western blot analysis. RAD001 (100 nM–1 *μ*M) was added 24 h before initiation of CXCL12 treatment. SN12C and A498 cells were serum-starved for 6 h and treated with the AMD3100 (5 *μ*M) 1 h before their exposure to CXCL12 (100 ng/ml for 10 min). Total P70S6 kinase, 4EBP1, ERK1/2 and P38 proteins were used for normalization. The experiments were repeated three times. Representative data from one of three experiments. Relative optical density of the bands in arbitrary units is represented for p-P70S6K, p4E-BP1, p-ERK1/2 and p-P38 reported, respectively, to the total P70S6K, 4EBP1, ERK1/2 and P38 (Supplementary Figure 1b)

To evaluate the CXCR4-dependent mTOR induction in renal cancer, two human renal cancer cell lines, A498, high CXCR4-expressing cells, and SN12C cells, low expression of CXCR4 (Supplementary Figure 1a), were analyzed. In Figure 1, SN12C and A498 cells were treated with CXCL12 (100 ng/ml) and with the CXCR4 inhibitor, AMD3100, in the presence of the mTOR inhibitor, RAD001, at different concentrations (100 nM and 1 *μ*M). CXCL12 induced the mTOR targets p-P70S6K and p-4EBP1 in SN12C and A498 cells and the CXCR4 antagonist, AMD3100, inhibited this induction, suggesting that CXCL12 signals on mTOR. Moreover, CXCL12 induced phosphorylation of ERK1/2 and P38; CXCL12-induced p-ERK1/2 was specifically inhibited by AMD3100, whereas CXCL12-induced p-P38 was not inhibited by AMD3100, suggesting that CXCR7 more than CXCR4 signals on P38 in agreement with previous report describing AMD3100 as a possible CXCR7 agonist.^30^ As expected RAD001 (100 nM and 1 *μ*M) impaired CXCL12-mediated induction of the mTOR targets P70S6K and 4EBP1 and modestly affected ERK and P38 signaling, suggesting that mTOR inhibition is downstream of CXCR4.

### CXCL12–CXCR7 transduces through mTOR in human renal cancer cells

**Figure 2:**
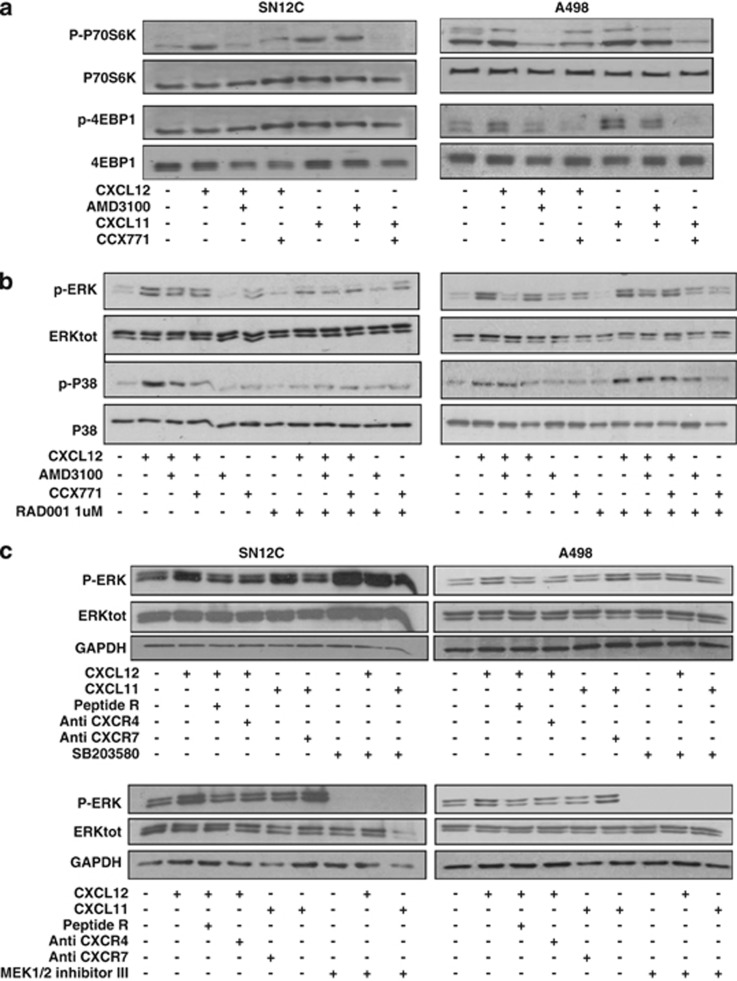
CXCR7 signals through the mTOR signaling pathway in RCC. (**a**) The SN12C and A498 human renal cancer cell lines were stimulated with CXCL11 (100 ng/ml) or CXCL12 (100 ng/ml) for 10 min AMD3100 (5 *μ*M) or CCX771 (100 nM) were added 1 h before the induction with CXCL12 or CXCL11. pP70S6 kinase and p-4EBP1 protein expressions were measured using western blot analysis. Representative data from one of three experiments. (**b**) CXCL12-induced ERK1/2 and P38 activation was measured in SN12C and A498 cells. Inhibitor of mTOR (RAD001; 1 *μ*M) was added 24 h before initiation of CXCL12 treatment. Cells were stimulated with CXCL12 for 10 min with or without AMD3100 (5 *μ*M) or CCX771 (100 nM) as above and analyzed using western blot analysis. Total ERK1/2 and P38 proteins were used for normalization. Representative data from one of three experiments. (**c**) CXCL12/CXCL11 -induced ERK1/2 activation was measured in SN12C and A498 cells treated with or without Peptide R (10 *μ*M), anti-CXCR4 (12G5) (10 *μ*g/ml) and anti-CXCR7 (11G8) (10 *μ*g/ml) 1 h before their exposure to CXCL12 or CXCL11 (100 ng/ml for 10 min). P38 inhibitor SB203580 (20 *μ*M) and ERK1/2 inhibitor MEK1/2 inhibitor III (5 *μ*M) were added 1 h before the initiation of CXCL12/CXCL11 treatment. Total ERK1/2 and GAPDH were used for normalization. Representative data from one of three experiments. Relative optical density of the bands in arbitrary units is represented for p-P70S6K, p-4EBP1, p-ERK1/2 and P-p38 reported, respectively, to the total P70S6K, 4EBP1, ERK1/2 and P38 (Supplementary Figures 2a and b)

To define the role of CXCR7 on the mTOR pathway, SN12C and A498 cells were stimulated with CXCL12 (100 ng/ml) or with the exclusive CXCR7 ligand CXCL11 (100 ng/ml) in the presence of a CXCR7 inhibitor, CCX771. As shown in Figure 2a, CXCL12 and CXCL11 induced p-P70S6K and p-4EBP1. Whereas CXCL12-mediated induction of p-P70S6K and p-4EBP1 was inhibited by AMD3100 and CCX771, CXCL11-mediated induction of p-P70S6K and p-4EBP1 was inhibited only by the specific CXCR7 inhibitor CCX771, suggesting that mTOR inhibition is also downstream of CXCR7. The CXCR7 signal transduction pathway is still controversial but certainly CXCR7 heterodimerizes with CXCR4 and potentiates the *β*-arrestin pathway as well as ERK1/2 and P38 signaling,^20^ thus the ERK1/2 and P38MAPK signaling pathway was investigated. Interestingly, CXCL12-mediated p-ERK1/2 and p-P38 were inhibited by the CXCR7 inhibitor CCX771 and RAD001 weakly affected the ERK1/2 and P38 signaling (Figure 2b). CXCL12-induced p-ERK1/2 was also inhibited by the treatment with Peptide R, a newly developed CXCR4 antagonist^31^ (10 *μ*M), by the anti-CXCR4 (12G5; 10 *μ*g/ml), whereas CXCL11-induced p-ERK1/2 was inhibited by anti-CXCR7 (11G8; 10 *μ*g/ml; Figure 2c). Interestingly, P38 inhibitor SB203580 did not affect CXCL11- or CXCL12-induced p-ERK1/2 activation, suggesting that P38 is not involved in the signal transduction of CXCR4 and CXCR7 on p-ERK1/2 whereas, as expected, the MEK1/2 inhibitor III blocked the p-ERK1/2 induction (Figure 2c).

**Figure 3:**
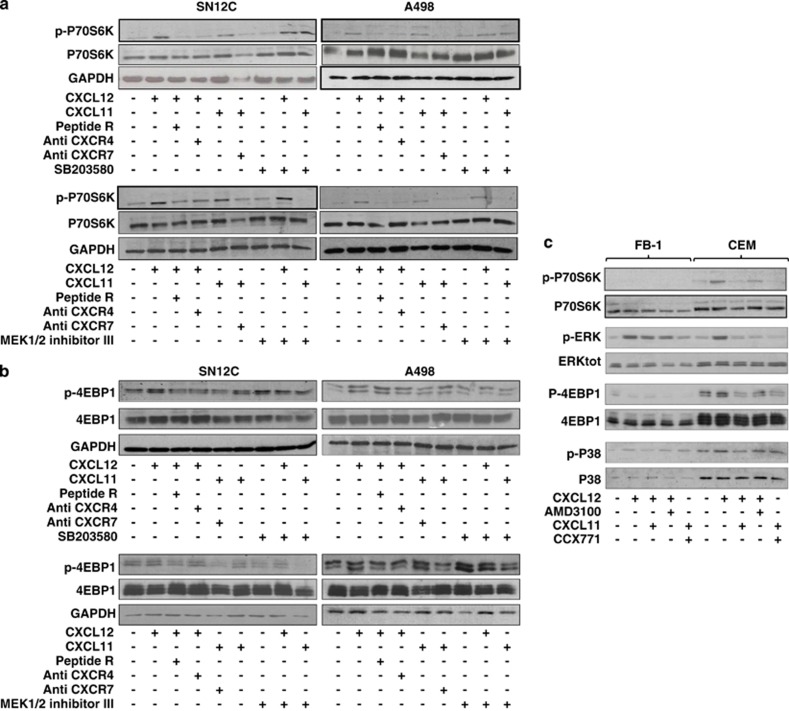
Effect of inhibitors for p38 and ERK kinases on the CXCR4–CXCR7–mTOR signaling pathway in RCC. (**a**) pP70S6 kinase protein expression was measured in A498 and SN12C cells using western blot analysis with or without Peptide R (10 *μ*M), anti-CXCR4 (12G5; 10 *μ*g/ml) and anti-CXCR7 (11G8; 10 *μ*g/ml) hour before their exposure to CXCL12 or CXCL11 (100 ng/ml for 10 min). Cultured RCC human cells were serum-starved, stimulated with the chemokines (100 ng/ml) for 10 min in the absence or presence of P38 inhibitor SB203580 (20 *μ*M) and ERK1/2 inhibitor MEK1/2 inhibitor III (20 *μ*M) for 1 h before CXCL12/CXCL11 treatment. Total P70S6 kinase and GAPDH were used for normalization. Representative data from one of three experiments. (**b**) p-4EBP1 protein expression was measured in A498 and SN12C cells using western blot analysis with or without Peptide R (10 *μ*M), anti-CXCR4 (12G5; 10 *μ*g/ml) and anti-CXCR7 (11G8; 10 *μ*g/ml) hour before their exposure to CXCL12 or CXCL11 (100 ng/ml for 10 min). P38 inhibitor SB203580 (20 *μ*M) and ERK1/2 inhibitor MEK1/2 inhibitor III (5 *μ*M) were added 1 h before the initiation of CXCL12/CXCL11 treatment. Cultured RCC human cells were serum-starved, stimulated with the chemokines (100 ng/ml) for 10 min in the absence or presence of P38 inhibitor SB203580 (20 *μ*M) and ERK1/2 inhibitor MEK1/2 inhibitor III (5 *μ*M) for 1 h before CXCL12/CXCL11 treatment. Total 4EBP1 kinase and GAPDH were used for normalization. Representative data from one of three experiments. (**c**) CXCL12/CXCL11-induced P70S6 kinase, 4EBP1, ERK1/2 and P38 proteins were measured in FB-1 and CEM cells. Cells were stimulated with CXCL12 or CXCL11 for 10 min with or without AMD3100 (5 *μ*M) or anti-CXCR7 (11G8; 10 *μ*g/ml) and analyzed using western blot analysis. Total P70S6K, 4EBP1, ERK1/2 and P38 were used for normalization. Representative data from one of three experiments

To further characterize the CXCR4–CXCL12–CXCR7 pathway in renal cancer cells, the induction of the mTOR targets P70S6K and 4EBP1 was evaluated in the presence of anti-CXCR4 (12G5), Peptide R and anti-CXCR7 (11G8). In Figure 3a, p-P70S6K was induced by CXCL12 and CXCL11 but inhibited by anti-CXCR4 (12G5), Peptide R and anti-CXCR7 (11G8). In Figure 3b, anti-CXCR4 (12G5), Peptide R and anti-CXCR7 (11G8) inhibited CXCL12/CXCL11-mediated induction of p-4EBP1. Interestingly, whereas P38 inhibitor (SB203580) did not affect mTOR induction, the MEK1/2 inhibitor III impaired CXCL11-dependent p-4EBP1and p-P70S6K, suggesting that CXCR7 could signal on mTOR through p-ERK1/2. Moreover, the induction of the mTOR pathway was evaluated in FB-1, human anaplastic thyroid cells, expressing extremely low levels of CXCR4 and CXCR7 and in CEM, human lymphoblastic T cells, expressing extremely high level of CXCR4 and low CXCR7. As shown in Figure 3c, CXCL12 and CXCL11 were not able to induce the mTOR targets p-P70S6K and p-4EBP1 in FB-1 cells, whereas in CEM cells they were induced and also regulated by specific inhibitors, suggesting that CXCR4 has a central role in mTOR induction. As CXCR7 was very low in CEM cells, CXCL11 was not able to activate neither the mTOR targets nor the CXCR7 targets, P38 and ERK1/2.

### CXCR4–CXCL12–CXCR7 and mTOR regulate cell migration in human renal cancer cells

**Figure 4:**
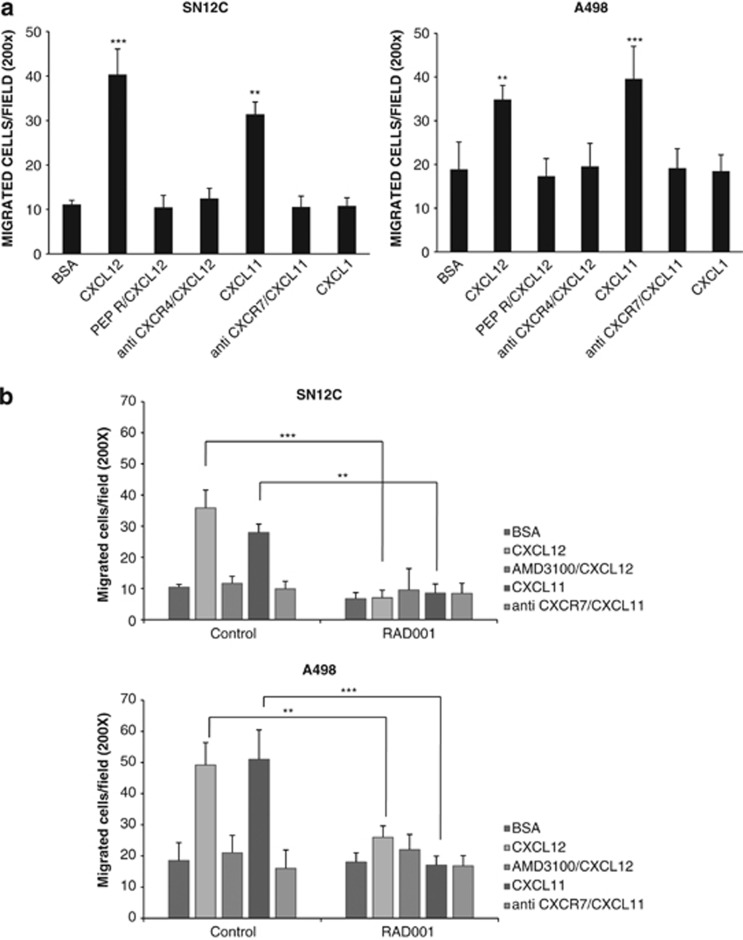
RAD001 blocks RCC migration CXCL12/CXCL11 induced. (**a**) CXCL12-dependent cell migration was examined in human RCC cell lines SN12C and A498 in 24-well plates. Cells (2.0 × 10^5^ cells/well) were placed in the upper chamber (8 *μ*m) in the presence of Peptide R (10 *μ*M), anti-CXCR4 (12G5; 10 *μ*g/ml) and anti-CXCR7 (10 *μ*g/ml). Cells migrated toward CXCL12 (100 ng/ml), CXCL11 (100 ng/ml) and CXCL1 (100 ng/ml) for 18 h. The chemokine CXCL1 demonstrated no effect on the migration of SN12c and A498 renal cancer cells. The cells were counted in 10 different consecutive high-power fields (magnification × 200). Each column represents the mean±S.D. (*n*=3). Statistical significances were calculated by Student's *t*-test. **P<0.001, ***P<0.001 *versus* BSA and relative inhibitor. (**b**) CXCL12/CXCL11-dependent cell migration was examined in human RCC cell lines SN12C and A498 in presence of RAD001 (100 nM). Cells were treated with RAD001 (100 nM) for 24 h and then cells (2.0 × 10^5^ cells/well) were placed in the upper chamber (8 *μ*m) in the presence of Peptide R (10 *μ*M), AMD3100 (5 *μ*M) and anti-CXCR7 (10 *μ*g/ml). Cells migrated toward CXCL12 (100 ng/ml) for 18 h. The cells were counted in 10 different consecutive high-power fields (magnification × 200). Each column represents the mean±S.D. (*n*=3). Statistical significances were calculated by Student's *t*-test. **P<0.001; ***P<0.0001 as specified by brackets

CXCL12 promotes cell motility and proliferation through CXCR4 and CXCR7.^24, 32, 33^ To investigate on CXCR4, CXCR7 and mTOR functions in A498 and SN12C cells, migration was evaluated in the presence of CXCR4–CXCR7ligand, CXCL12 and CXCL11 in the presence of anti-CXCR4 (12G5; 10 *μ*g/ml), anti-CXCR7 (11G8; 10 *μ*g/ml) and Peptide R (10 *μ*M). As shown in Figure 4a, SN12C and A498 cells migrated toward CXCL12 and CXCL11; the migration toward CXCL12 was inhibited by anti-CXCR4 and Peptide R, whereas CXCL11-induced migration was inhibited by anti-CXCR7 demonstrating that both chemokine receptors affected migration in renal cancer cells. To evaluate the role of mTOR in the migration, SN12C and A498 cells were pretreated with RAD001 (100 nM) for 24 h. RAD001 inhibited the CXCL12 and CXCL11 induced migration suggesting a central role for mTOR in CXCR4/CXCR7-mediated migration (Figure 4b). The effect of the CXCR4–CXCR7-unrelated chemokine, CXCL1, did not affect SN12C and A498 migration. Absence of nonspecific toxic effect of RAD001 was detected (Supplementary Figure 4).

**Figure 5:**
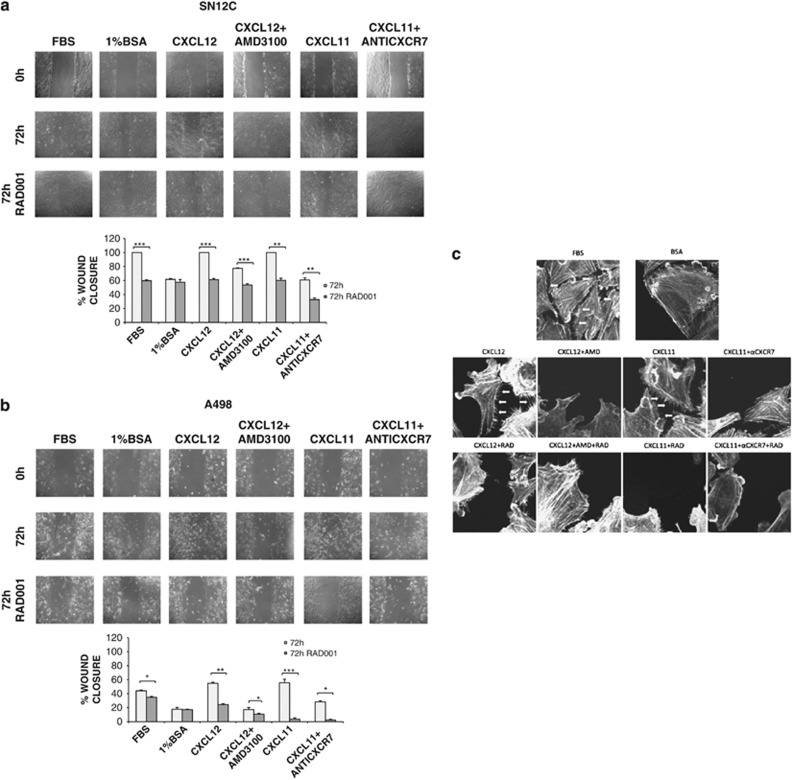
RAD001 blocks RCC wound closure and actin reorganization CXCL12/CXCL11 induced. (**a** and **b**) Delay in wound healing was evaluated in SN12C and A498 after 72 h in presence of RAD001 (100 nM) compared with CXCL12 or CXCL11 (100 ng/ml) with and without relative inhibitors AMD3100 (5 *μ*M) and anti-CXCR7 (10 *μ*g/ml). Confluent SN12C and A498 cells were scratched and treated with CXCL12 or CXCL11 (100 ng/ml) with and without anti-CXCR7 (10 *μ*g/ml) and AMD3100 (5 *μ*M) in presence or not of RAD001 for 72 h. Images were visualized and photographed with a digital camera (Canon) attached to microscope (Zeiss axiovert 40 C). The wound-healing area was measured using ImageJ 1.41 software (Bethesda, MD, USA). Data represent mean±S.D. for three different experiments. Statistical significances were calculated by Student's *t*-test. *P<0.05, **P<0.001, ***P<0.0001 as specified by brackets. (**c**) Inhibition of actin reorganization was examined in A498 with CXCL12 or CXCL11 (100 ng/ml) in presence of AMD3100 (5 *μ*M), anti-CXCR7 (10 *μ*g/ml) with and without RAD001 (100 nM) as specified. Actin was stained with Phalloidin TexRed. Images were acquired with confocal microscope (Zeiss LSM 540)

To confirm the role of mTOR and CXCR4/CXCR7 in cell motility, wound-healing experiments were carried out in SN12C and A498 cells. As shown in Figures 5a and b, respectively, CXCL12 and CXCL11 induced the wound closure that was impaired by CXCR4 antagonist AMD3100 and by anti-CXCR7 (11G8) or CXC771 (Supplementary Figure 5) at 72 h. RAD001 addition further inhibited the wound healing (lowest panel in Figures 5a and b). To evaluate whether the cytoskeleton could be target of CXCR4, CXCR7 and mTOR signal transduction, SN12C and A498 cells were treated with CXCL12 and CXCL11 and actin distribution was evaluated. CXCL12 and CXCL11 determined actin reorganization characterized by thin spikes at the cell periphery, after 72 h in SN12C (Figure 5c) and A498 (data not shown). AMD3100 and anti-CXCR7 (11G8) were able to prevent the actin modifications induced by CXCL12 and CXCL11, whereas further reduction of actin spikes was determined by RAD001 treatment. Interestingly, actin spikes at the cell periphery were prevented also by RAD001 treatment alone.

### CXCR4–CXCL12–CXCR7 and mTOR regulate cell growth in human renal cancer cells

**Figure 6:**
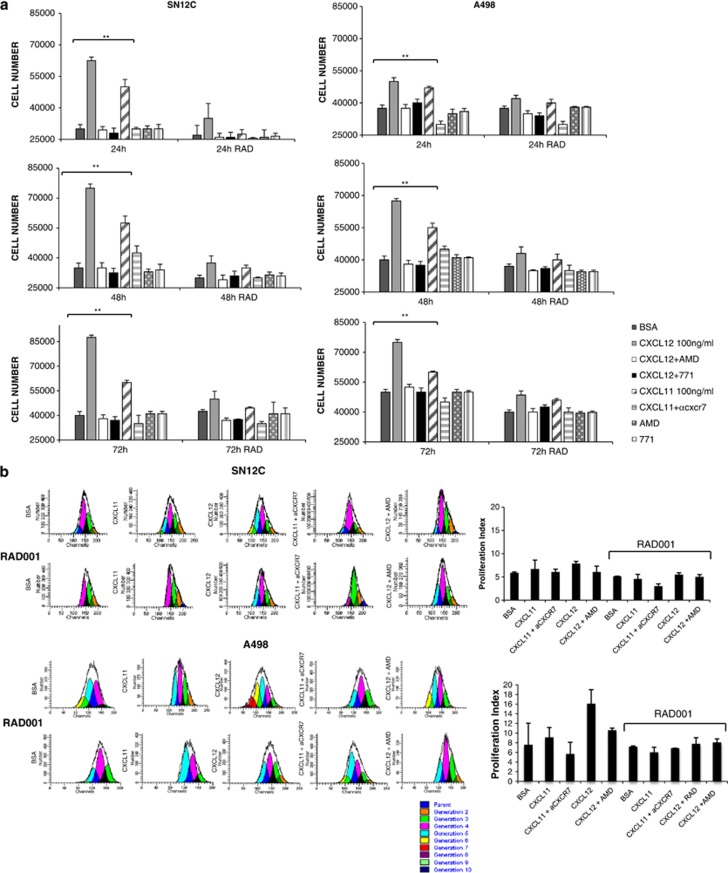
RAD001 blocks RCC proliferation CXCL12/CXCL11 induced. (**a**) SN12C and A498 cells were suspended in serum-free medium and cell growth was evaluated in the presence of CXCL12 (100 ng/ml), CXCL11 (100 ng/ml), AMD3100 (5 *μ*M) and anti-CXCR7 (10 *μ*g/ml) in presence of RAD001 (100 nM). Results are representative of three different experiments performed. Each column represents the mean±S.D. Statistical significances were calculated by Student's *t*-test. **P<0.001 CXCL12 *versus* BSA, CXCL12 *versus* AMD3100 or anti-CXCR7, CXCL11 *versus* BSA, CXCL11 *versus* anti-CXCR7. (**b**) CFSE-labeled human SN12C and A498 cells (1 × 10^5^ cells/ml) were treated with 1% BSA, AMD3100 (5 *μ*M), CXCL11 (100 ng/ml), CXCL12 (100 ng/ml), anti-CXCR7 (10 *μ*g/ml) with and without RAD001 (100 nM) at 37 °C. CFSE profiles of SN12C and A498 that were analyzed 24 h after CFSE labeling. CFSE staining was measured using flow cytometer and data were analyzed using the FACS Diva software. Results shown are representative of three experiments

As the CXCR4–CXCL12–CXCR7 axis and the mTOR pathway were described to regulate cell growth in renal cancer, we wished to evaluate whether targeting the two pathways might affect cell proliferation of SN12C and A498. In Figure 6a, the cell growth of A498 and SN12C cells was evaluated in the presence of CXCL11 or CXCL12, AMD3100 and anti-CXCR7 (11G8). SN12C (left panel) and A498 (right panel) cell growth was impaired by the CXCR4 and CXCR7 antagonists; the addition of RAD001 clearly reduced the CXCL12- and CXCL11-mediated cell growth. Comparable results were reported in the presence of CXCL11 or CXCL12, AMD3100, CXC771 (Supplementary Figure 6). Interestingly, the treatment with AMD3100, anti-CXCR7 (Figure 6a) or CXC771 (Supplementary Figure 6), in absence of CXCL12 or CXCL11, minimally impaired the SN12C and A498 cell growth. In Figure 6b, the effect of CXCL12, CXCL11 and the relative inhibitors was evaluated in the presence of RAD001 through the measurement of carboxyfluorescein diacetate succinimidyl ester (CFSE). As shown in Figure 6b, in SN12C cells the treatment with CXCL11 and CXCL12 induced cell growth that was inhibited by specific CXCR4 and CXCR7 inhibitors and further by RAD001 treatment. Comparable results were observed in A498 cells.

### Inhibition of the CXCR4–CXCL12–CXCR7 axis reinduces RAD001 sensitivity in RAD001-resistant renal cancer cell lines

**Figure 7:**
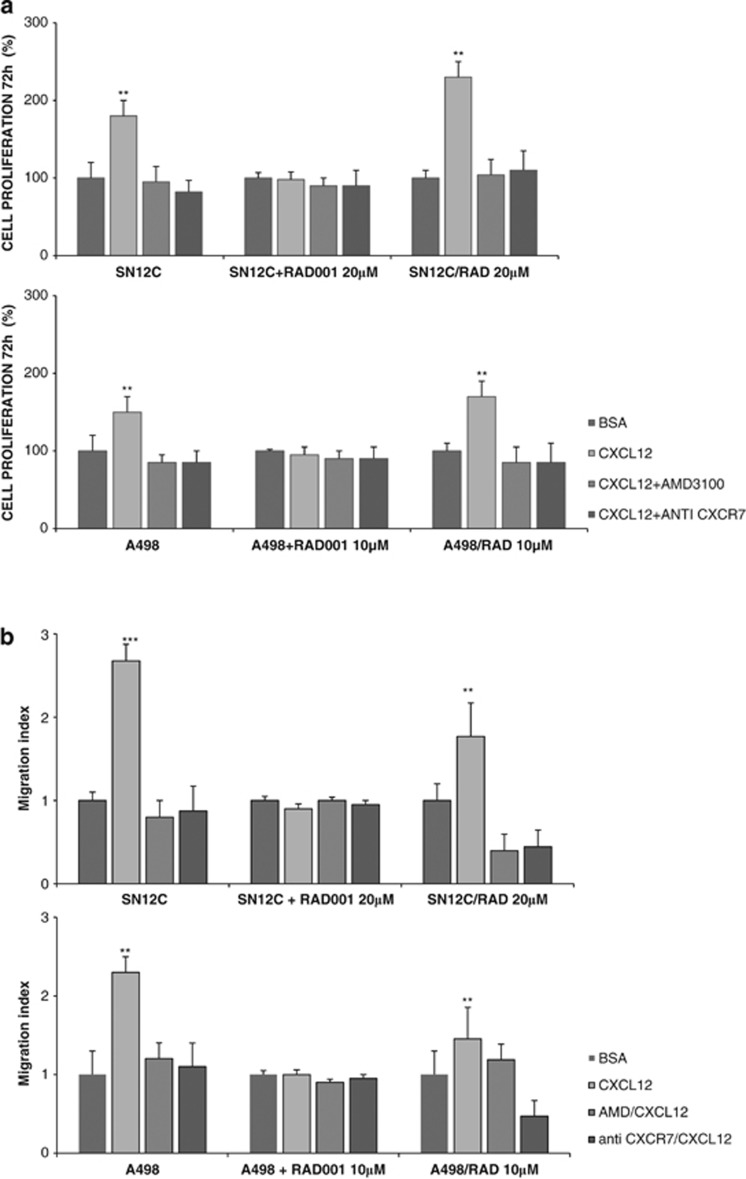
CXCR4–CXCL12–CXCR7 inhibitors reinduced sensitivity to RAD001 in RAD001-resistant renal cancer cell lines. (**a**) SN12C and SN12C/RAD 20 *μ*M cells and A498 and A498/RAD 10 *μ*M cells were suspended in serum-free medium and cell growth was evaluated in the presence of CXCL12 (100 ng/ml), AMD3100 (5 *μ*M) and anti-CXCR7 (10 *μ*g/ml) in presence or not of RAD001 (20 and 10 *μ*M). Results are representative of three different experiments performed. Each column represents the mean±S.D. Statistical significances were calculated by Student's *t*-test. **P<0.001. CXCL12 *versus* BSA, CXCL12 *versus* AMD3100 or anti-CXCR7. (**b**) CXCL12-dependent cell migration was examined in human RCC cell lines SN12C and SN12C/RAD 20 *μ*M, A498 and A498/RAD 10 *μ*M, in presence or not of RAD001 (20 or 10 *μ*M). Cells (2.0 × 10^5^ cells/well) were placed in the upper chamber (8 *μ*m) in the presence of AMD3100 (10 *μ*M) and anti-CXCR7 (10 *μ*g/ml). Cells migrated toward CXCL12 (100 ng/ml) for 18 h. The cells were counted in 10 different consecutive high-power fields (magnification × 200). Each column represents the mean±S.D. (*n*=3). Statistical significances were calculated by Student's *t*-test. **P<0.001, ***P<0.001 *versus* BSA and relative inhibitor

As CXCR4–CXCL12–CXCR7 axis regulated the mTOR pathway, it was speculated that CXCR4 and CXCR7 axis inhibition would enhance mTOR inhibitor efficacy and impair mTOR inhibitor drug resistance. RAD001-resistant A498 and SN12C sublines were generated (1–5–10 *μ*M—A498 and 1–5–20 *μ*M—SN12C). As shown in Figures 7a and b CXCL12 increased cell growth and induced cell migration in SN12C/RAD 20 *μ*M and A498/RAD 10 *μ*M. In SN12C and A498 RAD resistant cells the CXCL12 sensitivity was restored; CXCL12 increased cell growth in SN12C/RAD 20 *μ*M and A498/RAD 10 *μ*M cells of 2.4- and 1.8-fold, respectively, as compared to the CXCL12+RAD001 treatment of SN12C and A498 cells. Also CXCL12 induced migration was 1.8- and 1.5-fold higher in SN12C/RAD 20 *μ*M and A498/RAD 10 *μ*M cells compared to SN12C and A498 cells in the presence of CXCL12+RAD001. In SN12C/RAD 20 *μ*M and A498/RAD 10 *μ*M, concomitant treatment with CXCL12 and AMD3100 or anti-CXCR7 reduced CXCL12-induced cell growth and migration at the level of the RAD001-sensitive cell lines (Figures 7a and b). Moreover, the RAD001 IC_50_ values for RAD001-resistant SN12C cells were reduced in the presence of CXCR4 antagonists, AMD3100 or Peptide R (10 *μ*M; Table1). As shown in Table1, AMD3100 and Peptide R greatly enhanced the cytotoxicity of RAD001 for SN12C/RAD 20 *μ*M cells from 37 to 11 or 13.4 *μ*M, respectively.

## Discussion

To identify additional therapeutic opportunities in renal cancer, the crosstalk between the CXCR4/CXCL12/CXCR7 axis and the mTOR pathway was investigated in human renal cancer cells.

In SN12C and A498, the common CXCR4–CXCR7 ligand, CXCL12, and the exclusive CXCR7 ligand, CXCL11, activated mTOR through P70S6K and 4EBP1 targets and the induction was specifically inhibited by CXCR4 and CXCR7 antagonists. When CXCR4 and CXCR7 functions were evaluated, the effect of CXCR4, CXCR7 and mTOR inhibitors was additive in impairing migration and cell growth. Moreover, inhibition of the CXCR4–CXCL12–CXCR7 axis reinduced RAD001 sensitivity in resistant renal cancer cell lines.

To the best of our knowledge, this is the first time that the chemokine receptor CXCR7 was shown to activate mTOR in human renal cancer cells signaling through ERK and P38. CXCR4 and CXCR7 expression can differentially modulate the biological activity because of divergent activation pathways.^34^ In acute renal failure, CXCR7 but not CXCR4 was responsible for the CXCL12-induced renal progenitor cell survival.^24^ Presently, the exact function of CXCR7 is still controversial. Some studies evidence that CXCR7 activates PI3K and MAPK signaling controlling cell growth and survival in normal and tumor cells;^14, 15, 17, 18, 35, 36^ our previous observations showed that the expression of CXCR4 and CXCR7 predicted shorter disease-free survival in renal cancer patients.^10^ In this manuscript, CXCL12 activates CXCR4/CXCR7 signaling through p38 and ERK1/2 MAPK. The P38 induction was inhibited by CXCR7 inhibitor, CCX771, whereas it was not inhibited by AMD3100, a CXCR4 antagonist described as CXCR7 allosteric agonist^30^ confirming that CXCR7 signals through ERK1/2 and P38. CXCR7 is a fully signaling receptor independent from G proteins;^13^.^35^ when the ligand (CXCL11 or CXCL12) binds to the appropriate receptor, CXCR7 binds to *β*-arrestin.^22, 34, 36^ CXCL12 induces CXCR7/CXCR4 heterodimer formation, recruits *β*-arrestin and activates migration via the *β*-arrestin pathway, including the ERK1/2, p38 MAPK and SAPK pathways.^20^ CXCL12 and CXCL11 induced the mTOR targets p-P70S6K and p-4EBP1; whereas the CXCL12 induction was inhibited by AMD3100 and CCX771, CXCL11-mediated inductions were inhibited only by the specific CXCR7 inhibitor, suggesting that CXCR7 signals on mTOR. Interestingly, CXCL12-mediated induction of p-ERK1/2 and p-P38 was inhibited by the CXCR7 inhibitor CCX771 and was only minimally affected by RAD001. CXCL12-mediated p-ERK1/2 was also inhibited by the treatment with Peptide R, a newly developed CXCR4 antagonist,^31^ by the anti-CXCR4 while the CXCL11-induced p-ERK1/2 was inhibited by anti-CXCR7. Interestingly, P38 inhibitor, SB203580, did not affect the CXCL11- or CXCL12-induced p-ERK suggesting that p-ERK induction is independent of p38, whereas, as expected, the MEK1/2 inhibitor III blocked the P-ERK induction. Conversely, whereas P38 inhibitor SB203580 did not affect the mTOR induction, the MEK1/2 inhibitor III impaired the CXCL11-dependent p-4EBP1 and p-P70S6K, suggesting that CXCR7 signals on mTOR through p-ERK.

RAD001 is an oral inhibitor of mTOR indicated for patients with advanced RCC whose disease has progressed on VEGF-targeted therapy. It was recently approved by the FDA for hormone receptor-positive, HER2/neu-negative advanced breast cancer, for the treatment of well- or moderately differentiated neuroendocrine tumors of pancreatic origin in adults with progressive disease.^37, 38^ To date, mTOR inhibitors have shown only modest efficacy in tumors in which they were expected to provide important benefits and patients develop resistance to therapy and progress.^39^ Resistance to therapy may occur either through intrinsic or extrinsic mechanisms. The mechanisms of intrinsic resistance include the presence of KRAS or BRAF mutations, loss of phosphatase and tension homolog deleted on chromosome ten, low cellular levels of p27 or 4EBP1 and overexpression of eIF4E.^39^ More is known about the mechanisms involved in extrinsic resistance, also known as evasive, or adaptive resistance of antiangiogenic therapies. mTORC1 inhibition has been reported to induce AKT activation^39^ and it is possible that inhibition of mTORC1 with rapalogs shifts the equilibrium to increased mTORC2 activity, leading to AKT Ser 473 phosphorylation and subsequent activation. Preclinical studies in non-Hodgkin lymphoma confirmed such rapamycin-induced mTORC2-mediated activation of Akt independently of PI3K signaling.^40, 41^ A third proposed mechanism of resistance comprises activation of a positive loop promoting survival pathways such as PI3K/AKT, ERK/MAPK, PIM kinases and PDK.^39^ Crosstalk between CXCR4–CXCL12 and PI3K/mTOR was previously described in peritoneal disseminated gastric cancer cells,^26^ pancreatic cancer cells^29^ and human T-cell leukemia.^27^ Rapamycin inhibited CXCL12-mediated activation of PI3K/mTOR and CXCL12-dependent cell migration in human gastric cancer cells.^28^ In pancreatic cancer, baseline intratumoral CXCL12 gene expression correlated with temsirolimus resistance in explant models. Moreover, CXCL12 treatment resulted in CXCR4-mediated PI3-kinase-dependent p70S6K phosphorylation on exposure to temsirolimus. Combinatorial therapy with AMD3465, CXCR4 small-molecule inhibitor and temsirolimus resulted in effective tumor growth inhibition to overcome temsirolimus resistance.^29^ Here we demonstrated that the CXCL12–CXCR4 axis signals on mTORC1 in renal cancer cell lines and that the mTORC1 inhibitor, RAD001, inhibits CXCL12-induced migration, wound healing and cell growth such as demonstrated with the involvement of mTORC1 and mTORC2 in tumor cell motility and cancer metastasis.^42, 43^ The entire CXCR4–CXCL12–CXCR7 axis, including CXCR7, activated the mTOR pathway and stimulated cell migration in human A498 and SN12C renal cancer cells, whereas RAD001 impaired the chemotactic responses CXCL12 induced. Moreover, targeting the CXCR4–CXCL12–CXCR7 pathway with antagonists (AMD3100 or Peptide R and anti-CXCR7) reinduced RAD001 sensitivity in SN12C- and A498-RAD001-resistant renal cancer cells. Therefore, mTOR inhibitors may be coupled with the CXCR4–CXCL12–CXCR7 axis inhibitors in renal cancer patients to prevent and/or overcome the mTOR drug resistance. AMD3100 is the only CXCR4-targeted market drug indicated for hematopoietic stem cell mobilization in poor mobilizer patients,^44, 45^ nevertheless other CXCR4 antagonists are in clinical development.^46^ A new class of CXCR4 antagonists efficient in solid tumors was recently developed,^31^ whereas CXCR7 inhibitors such as CCX733 or CCX771 have been utilized to study CXCR7 function and signaling and might improve anticancer treatment.^35^ In conclusion, this is the first report on the concomitant involvement of CXCR4 and CXCR7 receptors both transducing on the mTOR pathway, affecting progression and spreading of human renal cancer cells suggesting that targeting CXCR4, CXCR7 and mTOR may improve therapeutic efficacy and prevent mTOR-targeting agents' resistance.

## Materials and Methods

### Cell lines

Human renal carcinoma cell lines A498 and SN12C and human T-leukemia cell line CCRF-CEM were obtained from NCI Frederick Cancer DCTD Tumor/Cell Line Repository. Human anaplastic thyroid cancer cell line, FB-1, was kindly provided by Professor Melillo (University of Naples, Federico II, Italy). Cell lines identified were confirmed by STR DNA typing at the Institute for Cancer Research (IST), Genova, Italy. Cell lines were cultured under standard culture condition at 37 °C in a humidified atmosphere of 5% CO_2_. The RAD001-resistant A498 and SN12C cell lines (A498/RAD and SN12C/RAD) were established by exposing the RAD001-sensitive A498 and SN12C parental cells to RAD001 from 1 nM to the final 1, 5, 10 or 20 *μ*M over a 12-month period. Resistant cells were routinely cultured in complete medium containing relative concentration of RAD001.

### Reagents

The CXCR4 inhibitor, AMD3100, was obtained from Sigma (St. Louis, MO, USA). The high-affinity CXCR7 ligand, CCX771, was a gift from ChemoCentryx^11, 18^ and used as reported below. The specific CXCR4 antagonist, Peptide R, was developed in our laboratory.^31^ RAD001 (Novartis, Charleston, SC, USA) provided by Professor Caraglia (Second University of Naples) used at 100 nM and 1 *μ*M. P38 inhibitor SB203050 and MEK1,2 inhibitor were from Calbiochem (San Diego, CA, USA).

### Antibodies

Rabbit monoclonal antibodies for p44/42 MAPK (Erk1/2), phospho-p44/42 MAPK (Erk1/2; T202/Y204), p70S6K1, phospho p70S6K1(Thr 389), phospho-S6K (T389), anti-4EBP1, anti-phospho 4EBP1, phospho P38MAPK (T180/Y182), P38 MAPK were from Cell Signaling Technology Inc. (Danvers, MA, USA) and mouse polyclonal antibodies for GAPDH were from Santa Cruz Biotechnology, Santa Cruz, CA, USA. Secondary antibodies include goat anti-rabbit-HRP (Jackson ImmunoResearch, West Grove, PA, USA) and goat anti-mouse-HRP (Santa Cruz Biotechnology). The anti-CXCR7 monoclonal antibody CXCR7/RDC-1 (Clone 11G8) and anti-CXCR4 monoclonal antibody human CXCR4 (Clone 12G5) were from R&D Systems (Minneapolis, MN, USA).

### Cytotoxicity assay

Cells were plated in 96-well plates (1500 cells/well) and 3-day cytotoxicity assays were performed using the SRB assay. Cells were incubated in quadruplicate in varying concentrations of RAD001 and 10uM AMD3100 or Peptide R for 72 h.

### Proliferation assay

SN12C and A498 cells were plated into six-well plates at a density of 25 × 10^4^ cells/well in duplicate. After 24 h, RAD001 (100 nM), AMD3100 (5 *μ*M), anti CXCR7 (10 *μ*g/ml) or a combination of both were added. Cells were incubated for 24–48–72 h at 37 °C in a humidified atmosphere containing 5% CO_2_ and counted using a hemocytometer.

### CFSE proliferation assay

A CFSE stock (10 mM in DMSO; Invitrogen, Merelbeke, Belgium) stored at −20 °C was thawed and diluted in DMSO.^46, 47^ A498 and SN12C cells were incubated in PBS for staining at 1 × 10^5^ cells/ml and with CFSE (final concentration: 5 *μ*M) for 15 min at 37 °C in the dark. Cells were washed and reincubated in culture medium for 30 min to stabilize the CFSE staining. After a final wash step, cells were incubated in culture medium 1% BSA with CXCL12 (100 ng/ml), CXCL11 (100 ng/ml), anti-CXCR7 (10 *μ*g/ml), AMD3100 (5 *μ*M) and RAD (100 nM). The cultures were incubated at 37 °C in a 5% CO_2_ incubator for 24 h. The samples were analyzed on FACSCanto II (Becton Dickinson, San Diego, CA, USA). From each sample, a minimum of 100 000 events was collected and analyzed using the FACS Diva software version 6.1.1 (Becton Dickinson, Mississauga, ON, Canada).

### Immunoblotting

Cells were lysed in a whole-cell buffer containing protease and phosphatase (10 mM NaF, 10 mM Na-pyrophosphate, 1 mM Na3VO4) inhibitors, then the supernatants were obtained using centrifugation at 4 °C, 14 000 r.p.m. for 15 min. Total protein (50 *μ*g) was separated using SDS-PAGE gel 10%. The signal was revealed through chemoluminescent detection kit (ECL detection kit, GE Healthcare, Milan, Italy).

### Cell motility/chemotaxis

Migration was assayed in 24-well transwell chambers using inserts (8-*μ*m pore membrane). Membranes were pre-coated with collagen and fibronectin. Cells were placed in the upper chamber (2.0 × 10^5^ cells/well) in DMEM containing 0.5% BSA (migration media), and 100 ng/ml CXCL12 was added to the lower chamber. After 18-h incubation, cells on the upper surface of the filter were removed using a cotton wool swab. Cell migration was quantified by determining the number of cells that had migrated into the filters. MDA-MB-231 human breast cancer cells were used as positive control. The cells were counted in 10 different consecutive high-power fields (magnification × 200).

### Wound-healing assay test and actin staining

Wound-healing test was performed according to the previously reported and standardized protocol.^48, 49^ SN12C and A498 cells were seeded on glass overslips in a 10-am tissue colture dish and grown to confluent cell monolayer. Afterward the coverslips were transferred to a 24-well plate and a small area was then scratched using a 200-*μ*l pipette tip, and the cells were then rinsed with PBS to remove the loosen debris of the cells. Media with FBS 10%, BSA 1%, CXCL12, CXCL11 with and without inhibitors were replaced and the plates were incubated at 37 °C and 5% CO_2_. The distance between two layers of cells that was scratched by pipette tip was then inspected microscopically at 0, 24, 48 and 72 h, respectively. As the cells migrate to fill the scratched area, images were captured by a digital camera (Canon) attached to microscope (Zeiss axiovert 40 C, Carl Zeiss MicroImaging, Inc., Jena, Germany). The experiments were performed in triplicate.

Actin staining was performed as before.^50, 51^ Briefly after the wound-healing assay, cells were fixed in 3% paraformaldehyde and permeabilized in 0.2% Triton after extensive washing in PBS. Phalloidin TexRed (Sigma) was used to stain F actin for 45 min in a dark chamber. After PBS washing, coverslips were mounted, analyzed and acquired using confocal microscope (Zeiss LSM 540).

### Statistical analysis

The values given are means±S.D. Student's *t*-test was used for comparing the means and differences with a *P*-value of <0.05 were considered significant.

## Figures and Tables

**Table 1:** RAD001 sensitivity in RAD001-resistant SN12C cell lines

**Cell line**	**RAD001 IC**_**50**_ **(*μ*M)**	**RAD001+peptide R IC**_**50**_ **(*μ*M)**	**RAD001+AMD3100 IC**_**50**_ **(*μ*M)**
SN12C	5.5±0.03	4.8±0.03	4.3±0.02
SN12C RAD, 1 *μ*M	5.8±0.03	2.3±0.03	4.6±0.02
SN12C RAD, 5 *μ*M	13.6±0.03	10.9±0.01	9.4±0.02
SN12C RAD, 20 *μ*M	37±0.02	11±0.03	13.4±0.01

Peptide R (10 *μ*M) or AMD3100 (10 *μ*M)

## Abbreviations

mTOR: Mammalian target of rapamycin
RCC: renal cell carcinoma
mRCC: metastatic renal cell carcinoma
CXCR4: CXC chemokine receptor 4
CXCR7 or RDC1: CXC chemokine receptor 7
CXCL12 or SDF-1: CXC chemokine ligand 12
CXCL11: CXC chemokine ligand 11
CXCL1: CXC chemokine ligand 1; RAD001 or RAD
Everolimus; 4EBP1: eukaryotic initiation factor 4E-binding protein 1
p70S6K or S6K: 70-kDa S6 protein kinase
ERK: extracellular signal-regulated protein kinase
PI3K: phosphatidylinositol 3-kinase
MEK: mitogen-activated protein kinase kinase
VHL: von Hippe–Lindau
HIF: hypoxia-inducible factor
GPCR: G protein-coupled receptor
CFSE: carboxyfluorescein diacetate succinimidyl ester
FACS: fluorescence-activated cell sorting

## Supplementary Material

Supplementary Figure 1A

Supplementary Figure 1B

Supplementary Figure 2A

Supplementary Figure 2B
