# Supplementary figures and images for: CXCR4 and CXCR7 transduce through mTOR in human renal cancer cells
Source: Cell Death Dis. 2014 Jul 3;5(7):e1310–. doi: 10.1038/cddis.2014.269 (PMC4123065; doi:10.1038/cddis.2014.269)

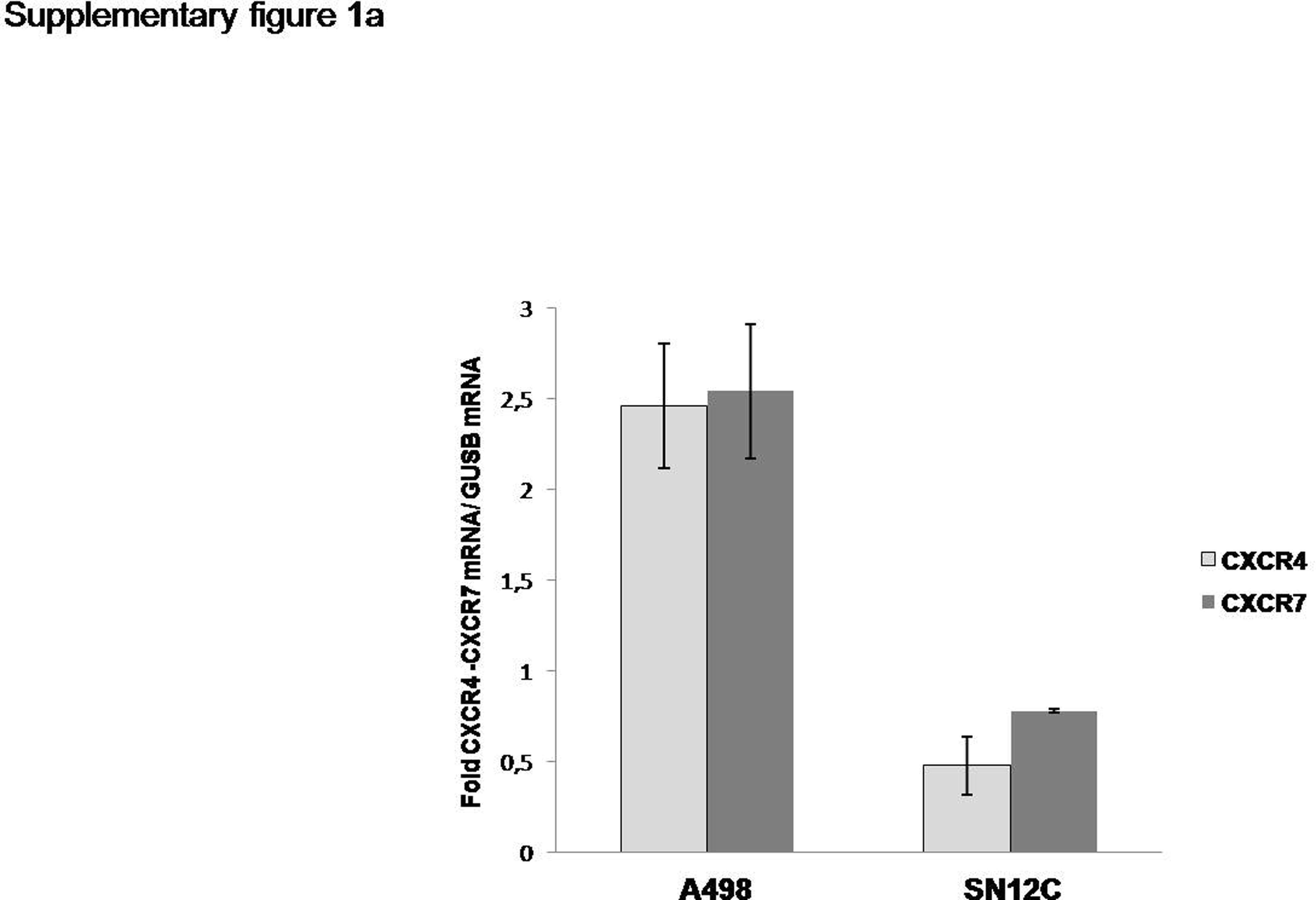

Supplement: Supplementary Figure 1A [file cddis2014269x1.tif]

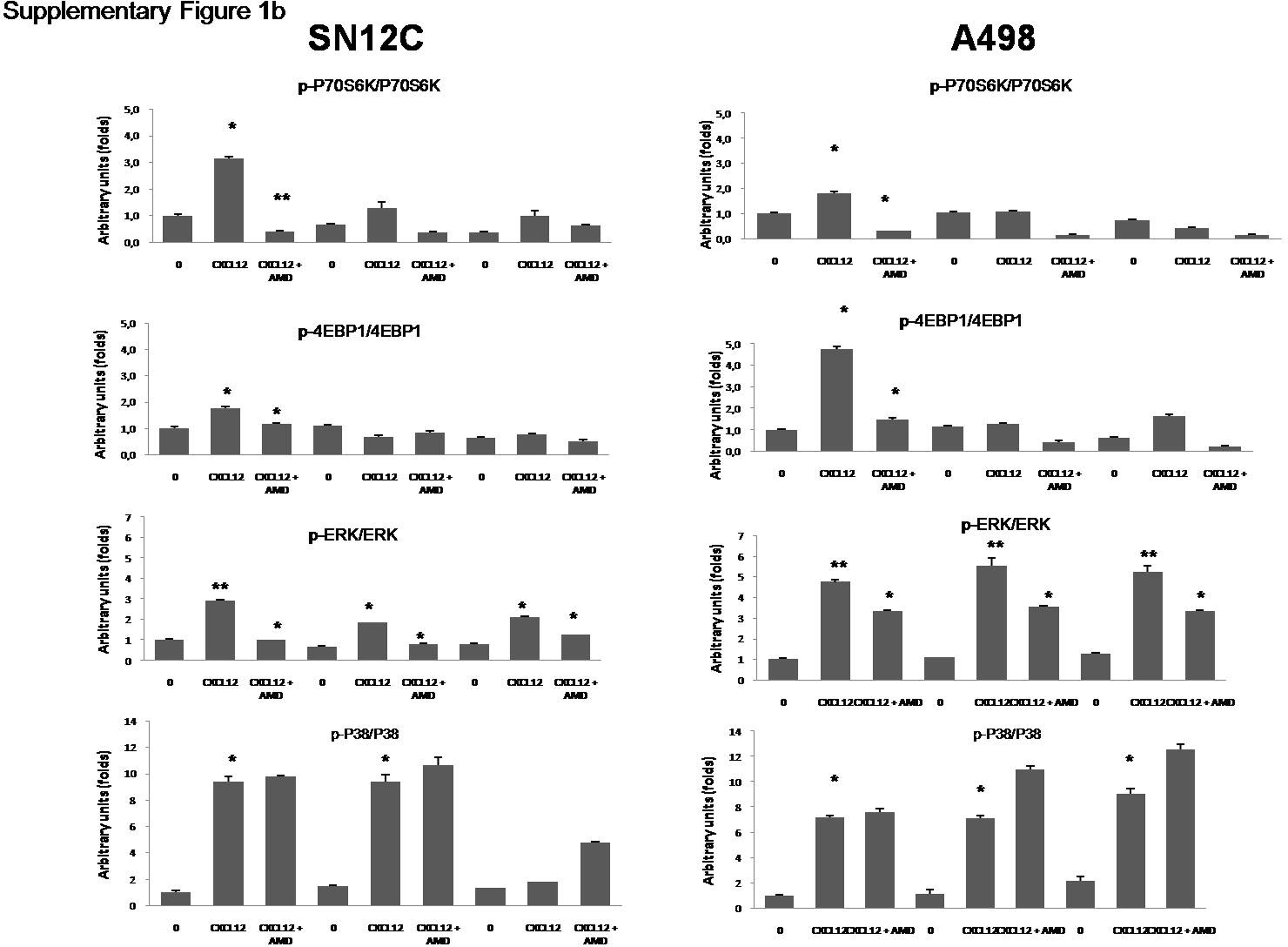

Supplement: Supplementary Figure 1B [file cddis2014269x2.tif]

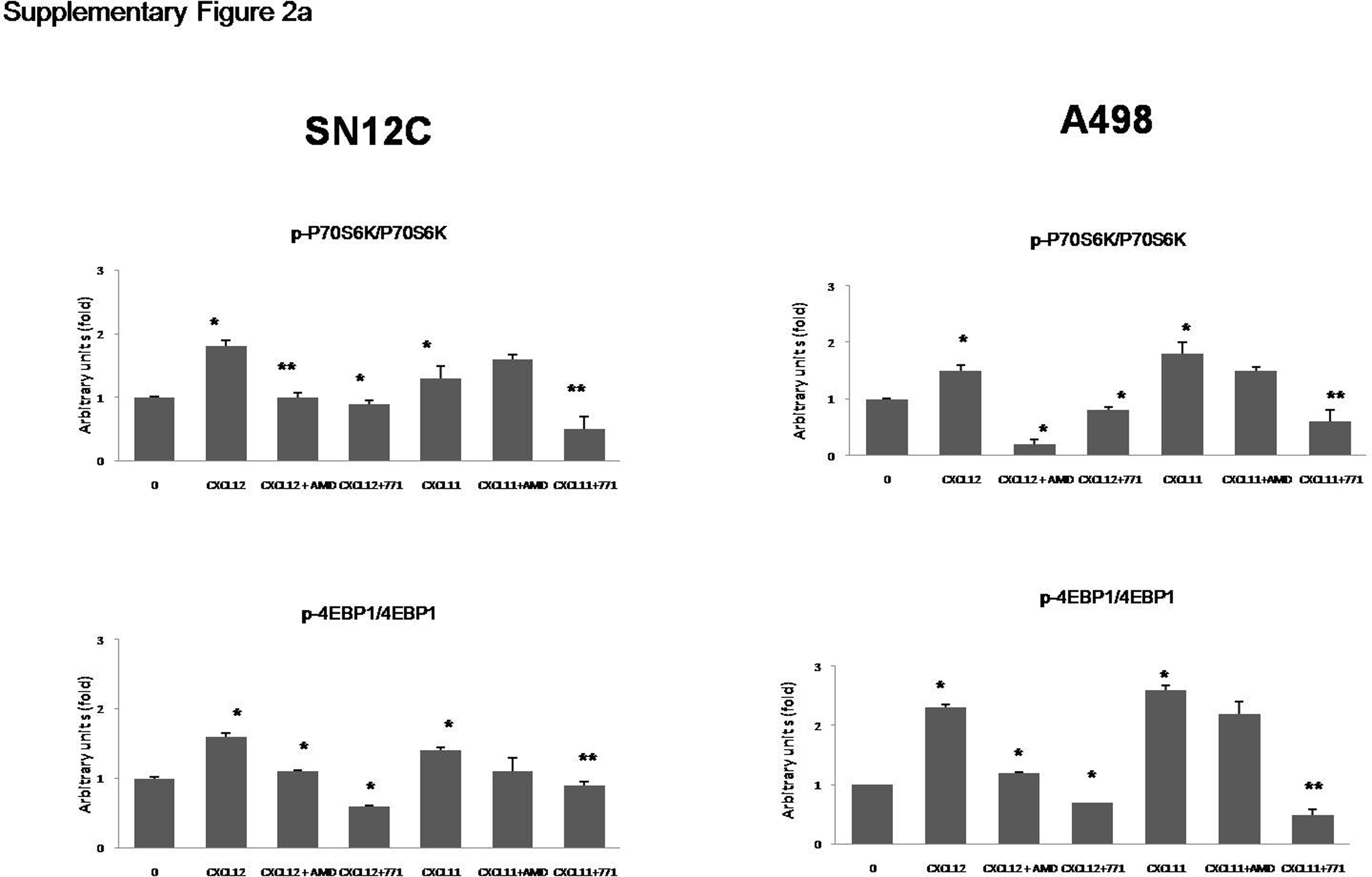

Supplement: Supplementary Figure 2A [file cddis2014269x3.tif]

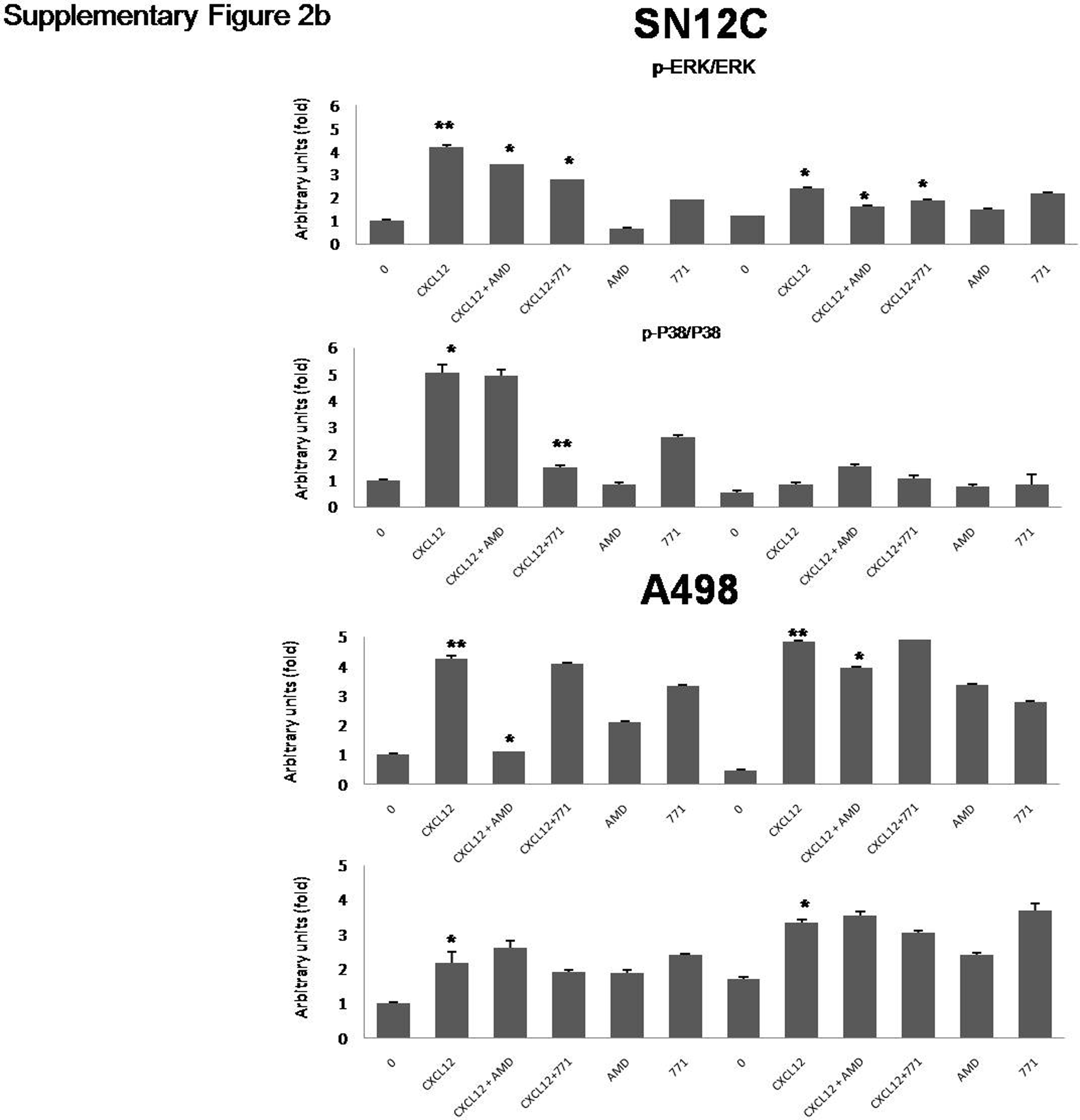

Supplement: Supplementary Figure 2B [file cddis2014269x4.tif]

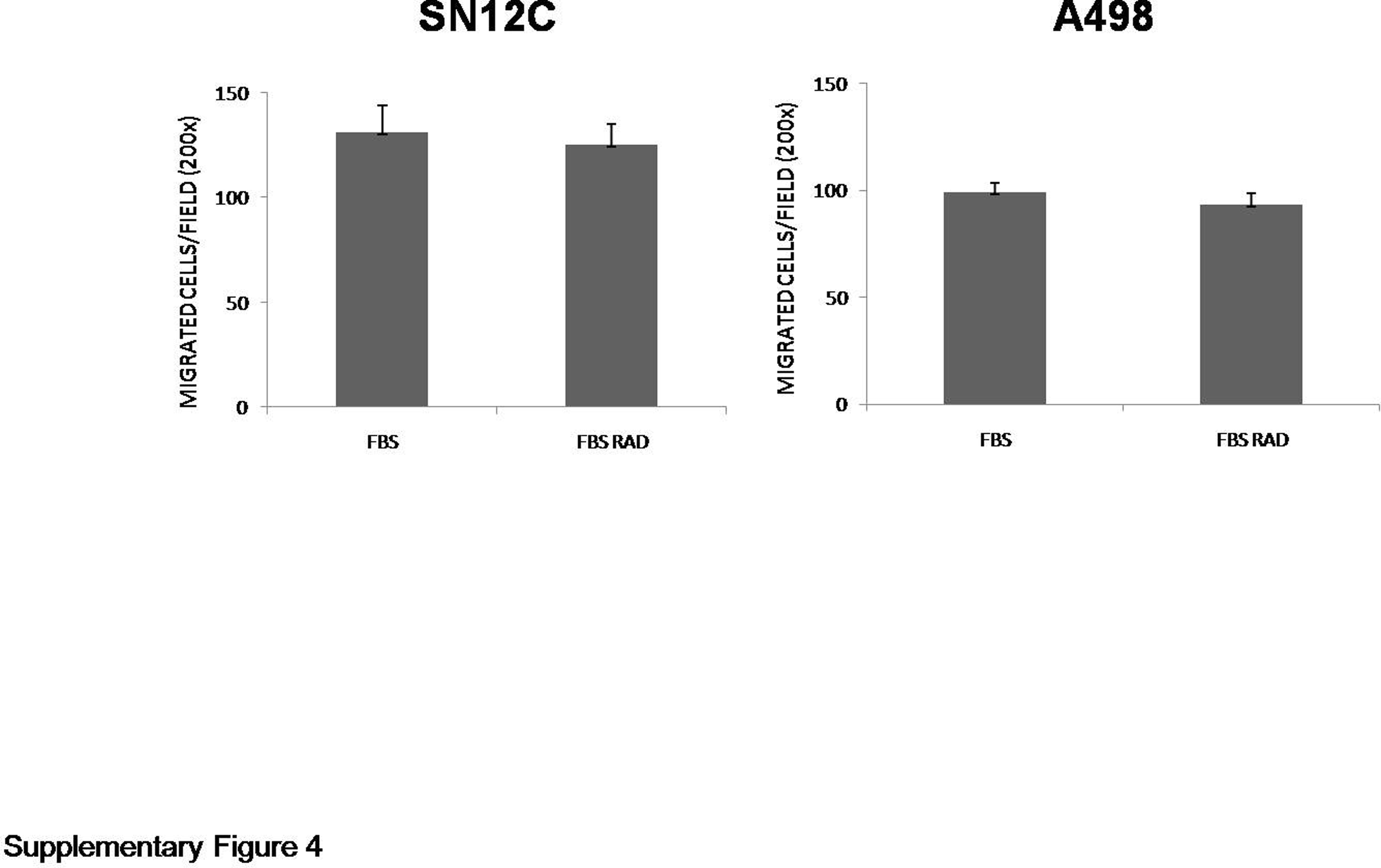

Supplement: Supplementary Figure 4 [file cddis2014269x5.tif]

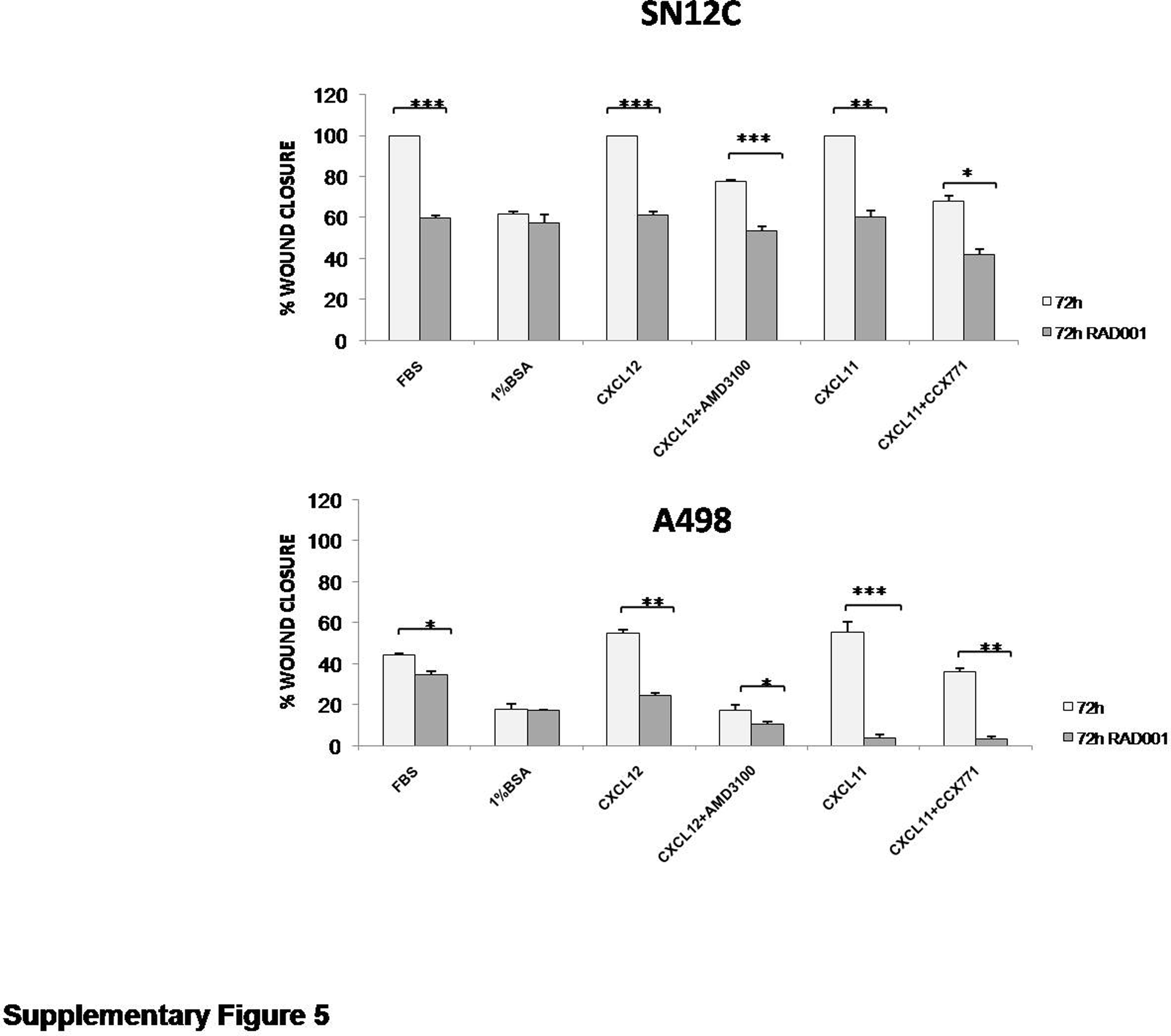

Supplement: Supplementary Figure 5 [file cddis2014269x6.tif]

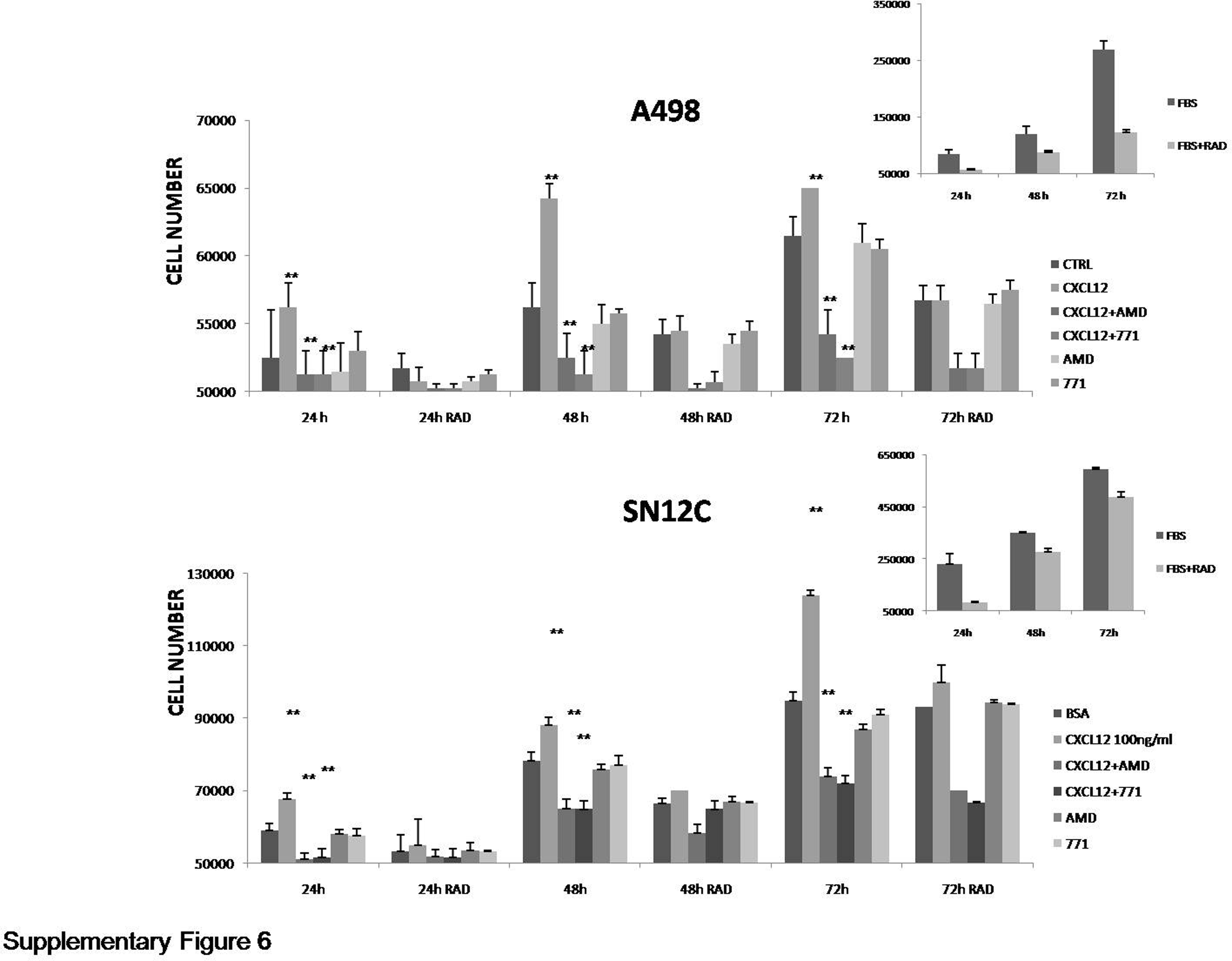

Supplement: Supplementary Figure 6 [file cddis2014269x7.tif]
