# Supplementary material for: CXCR4 and CXCR7 transduce through mTOR in human renal cancer cells
Source: Cell Death Dis. 2014 Jul 3;5(7):e1310–. doi: 10.1038/cddis.2014.269 (PMC4123065; doi:10.1038/cddis.2014.269)
Supplement: Supplementary Figure Legends [file cddis2014269x8.doc]

**Supplemental figure 1. CXCR4 and CXCR7 expression in RCC human cell lines.** (a) mRNA levels of CXCR4 and CXCR7 in SN12C and A498 human renal cancer cell lines. Basal levels CXCR4 and CXCR7 (normalized to GUSB levels) are analyzed by real-time PCR. Mean ± SD of three independent experiments are shown.(b) The SN12C and A498 human renal cancer cell lines were stimulated with CXCL12 (100 ng/ml) and p-P70S6 kinase, p-4EBP1, p-ERK1/2 and p-P38 protein were measured in absence or presence RAD001(100 nM and 1 uM) by Western blot analysis. Relative optical density of the bands in arbitrary units is represented for pP70S6K, p4E-BP1, pERK1/2 and pP38 reported, respectively to the total P70S6K, 4E-BP1, ERK1/2 and P38. The results are expressed as mean±SD. Control without CXCL12 is considered as 1. *p<0.05 **p<0.01 versus the group with CXCL12 or the group without CXCL12.

**Supplemental figure 2.** **CXCL12 actives mTOR signaling pathway through CXCR4 in RCC.** (a) The SN12C and A498 human renal cancer cell lines were stimulated with CXCL11 (100 ng/ml) or CXCL12 (100ng/ml) for 10 minutes with or without AMD3100 (5µM) or CCX771 (100nM) and pP70S6 kinase and p4EBP1 protein expression were measured by Western blot analysis. Relative optical density of the bands in arbitrary units is represented for pP70S6K and p4E-BP1, respectively to the total P70S6K, 4E-BP1. The results are expressed as mean±SD. Control without CXCL12 is considered as 1. *p<0.05 **p<0.01 versus the group with CXCL12 or the group without CXCL12. (b) CXCL12-induced ERK1/2 and P38 activation was measured in SN12C and A498 cells in presence of RAD001 (1µM) with or without AMD3100 (5µM) or CCX771 (100nM) by Western blot analysis. Relative optical density of the bands in arbitrary units is represented for pERK1/2 and pP38 reported, respectively to the total ERK1/2 and P38. The results are expressed as mean±SD. Control without CXCL12 is considered as 1. *p<0.05 **p<0.001 versus the group with CXCL12 or the group without CXCL12.

**Supplemental figure 4**. **RAD001 blocks RCC migration CXCL12/CXCL11 mediated.** (a) Basal migration toward 10% FBS in the presence of RAD001(100nM) was examined in human RCC cell lines SN12C and A498. Cells (2.0 x10^5^cells/well) were placed in the upper chamber (8µm) 24-well plates. (b) CXCL12 dependent-cell migration was examined in human RCC cell lines SN12C and A498 in presence of RAD001 (100nM).Cells were treated with RAD001 (100nM) for 24 hours and then cells (2.0 x10^5^cells/well) were placed in the upper chamber (8µm) in the presence of Peptide R (10µM), AMD3100 (5uM) and CXC771 (100nM). Cells migrated toward CXCL12 (100ng/ml) for 18 hours. The cells were counted in ten different consecutive high power fields (magnification 200X). Each column represents the mean ± S.D. (n=3). Statistical significances were calculated by Student’s t-test. **p<0.001 ***p<0.0001 as specified by brackets.

**Supplemental figure 5**. **RAD001 blocks RCC wound closure CXCL12/CXCL11 mediated.** In the graph was shown % wound healing in SN12C and A498 after 72 hours in presence of RAD001 (100nM) compared with CXCL12 or CXCL11 (100ng/ml) with and without relative inhibitors AMD3100 (5uM) and CXC771 (100nM). For quantifications, the area covered by cells for 72 hours was measured using ImageJ. Data represent mean ± S.D for 3 different experiments. Statistical significances were calculated by Student’s t-test. *p<0.05, **p<0.001 ***p<0.0001 as specified by brackets.

**Supplemental figure 6**. **RAD001 blocks RCC proliferation CXCL12/CXCL11 mediated.** SN12C and A498 cells were suspended in serum-free medium and cell growth was evaluated in the presence of CXCL12 (100 ng/ml), CXCL11 (100ng/ml), AMD3100 (5µM) and CXC771 (100nM) in presence of RAD001 (100 nM). Results are representative of 3 different experiments performed. Each column represents the mean ± S.D. Statistical significances were calculated by Student’s t-test. **p<0.001 compared to BSA or CXCL12 alone. Insert: Cell growth in10% FBS in the presence of RAD001 (100 nM).
