# Supplementary material for: CXCR4 and CXCR7 transduce through mTOR in human renal cancer cells
Source: Cell Death Dis. 2014 Jul 3;5(7):e1310–. doi: 10.1038/cddis.2014.269 (PMC4123065; doi:10.1038/cddis.2014.269)
Supplement: Supplementary Information [file cddis2014269x9.doc]

**RNA Isolation and Real-Time Reverse Transcription-Polymerase Chain Reaction.** RNA was extracted with RNeasy protect mini kit (Qiagen), treated with RNAse-free Dnase I (Invitrogen-Life Technologies, Carlsbad, CA) to prevent DNA contamination. cDNA was synthesized using 200ng RNA and 100 U Superscript III (Invitrogen, Karlsruhe, Germany) and random hexamer primers (Invitrogen) according to the manifacturer’s instructions. Real Time-PCR was carried out using 2 µl of a 1:4 dilution of 20 µl cDNA reaction in a 25 µl final SYBR Green (Power SYBR Green master mix from Applied Biosystem) reaction mixture. An ABI Prism 7000 (Applied Biosystem) robocycler was used for the amplification. Cycling conditions of the PCR were as follows: initial denaturation (10 minutes at 95°C) followed by 40 cycles of denaturation (15 seconds at 95°C) and annealing (1 minute at 60°C). The gene-specific primers used for the amplification were as follows:

CXCR4: 5’-TGGGTGGTTGTGTTCCAGTTT-3’ (forward)

5’-ATGCAATACCAGGACAGGATGA-3’ (reverse);

CXCR7: 5’- GATTGCCCGCCTCAGAAC-3’ (forward)

5’- GCAGGACGCTTTTGTTGG-3’ (reverse);

GAPDH: 5’- CGACAGTCAGCCGCATCT-3’ (forward)

5’- CGCCCAATACGACCAAA-3’ (reverse)

Subsequently, CXCR4 and CXCR7 mRNA were quantified comparing their expression to GAPDH mRNA levels using the comparative Ct method (Schmittgen, T. D.; Livak, J. Analyzing real time PCR data by the comparative C_t_ method. *Nat*. *Prot*., 2008, *3*, 1101-1108). CXCR4 and CXCR7 primers were also tested on positive and negative controls in order to establish their specificity. At least triplicate samples were used in all Real Time PCR experiments.
